# Hormonal treatment for endometriosis associated pelvic pain

**Published:** 2011

**Authors:** Wu Shun Felix Wong, Chi Eung Danforn Lim

**Affiliations:** 1 School of Women’s and Children’s Health, Faculty of Medicine, University of New South Wales, Sydney, Australia.; 2 South Western Sydney Clinical School, Faculty of Medicine, University of New South Wales, Sydney, Australia.

**Keywords:** *Endometriosis associated pelvic pain*, *Medical treatments*, *Progestogen*, *Combined oral contraceptive pills*, *GnRH*

## Abstract

**Background:**

Endometriosis is a common gynecological problem associated with chronic pelvic pain.

**Objective:**

To evaluate the effectiveness of current hormonal treatments of endometriosis associated pain.

**Materials and Methods:**

Randomized Controlled studies identified from databases of Medline and Cochrane Systemic Review groups were pooled. 7 RCTs were recruited for evaluation in this review. Data from these studies were pooled and meta-analysis was performed in three comparison groups: 1) Progestogen versus GnRHa; 2) Implanon versus Progestogen (injection); 3) Combined oral contraceptive pills versus placebo and progestogen. Response to treatment was measured as a reduction in pain score. Pain improvement was defined as improvement ≥1 at the end of treatment.

**Results:**

There was no significant difference between treatment groups of progestogen and GnRHa (RR: 0.036; CI:-0.030-0.102) for relieving endometriosis associated pelvic pain. Long acting progestogen (Implanon) and Mirena are not inferior to GnRHa and depot medroxy progesterone acetate (DMPA) (RR: 0.006; CI:-0.142-0.162). Combined oral contraceptive pills demonstrated effective treatment of relieving endometriosis associated pelvic pain when compared with placebo groups (RR:0.321CI-0.066-0.707). Progestogen was more effective than combined oral contraceptive pills in controlling dysmenorrhea (RR:-0.160; CI:-0.386-0.066), however, progestogen is associated with more side effects like spotting and bloating than the combined contraceptive pills.

**Conclusion:**

Combined oral contraceptive pills (COCP), GnRHa and progestogens are equally effective in relieving endometriosis associated pelvic pain. COCP and progestogens are relatively cheap and more suitable for long-term use as compared to GnRHa. Long-term RCT of medicated contraceptive devices like Mirena and Implanon are required to evaluate their long-term effects on relieving the endometriosis associated pelvic pain.

## Introduction

Endometriosis is a common gynecological disease, found in 70% of patient with chronic pelvic pain (1). It is characterized by the presence and growth of endometrial tissue outside the uterine cavity (1). This condition is typically associated with infertility; dyspareunia and dysmenorrhea, with the latter being the most frequent complaint by women with endometriosis (2). 

The cyclic nature of pain associated with endometriosis is probably attributed to the response of endometrial tissue to cycling reproductive hormones particularly estrogen (3). 

Treatment of endometriosis associated pelvic pain includes both medical and surgical options. Current medical treatment options include combined oral contraceptive pills, progestogens, androgen hormone (e.g. Danazol) and gonadotrophin-releasing hormone analogues (GnRHa). Each treatment options have its own systemic side effects, leading to no definite cure for endometriosis. Associated pelvic pain frequently recurs once medications are stopped due to reactivation of ectopic endometrial implants.

Progestogen is most commonly used for treatment of endometriosis (3). There aredifferent types of progestogens including medroxy progesterone acetate and 19-nortestosterone. 

Their proposed mechanism of action is to stop endometrial proliferation and to induce regressive changes (3, 4). In a Cochrane review performed by Prentice, Desary and Bland (2009) (5), they noted that progestogen was an effective treatment for endometriosis-associated pain. 

However, the conclusion from their review was based on limited data. Eight studies were recruited and majority of the recruited studies had a relative small sample size. The mean number of patients recruited was 82. 

Additionally, with the advanced therapeutic development, apart from orally and intramuscularly administered forms of delivering progestogen, there are other forms such as intrauterine device (Mirena) and subcutaneous implant (Implanon). 

They provide long- term release of progestogens up to three to five years. Prentice, Desary and Bland (2009) did not include these long-term releases of progestogen in their review (5). 

This paper aims to compare and to determine the effectiveness of current hormonal treatments of endometriosis associated pelvic pain. Treatments options included are progestogens, GnRHa and combined contraceptive pills. They are compared as following: 

1. Progestogen versus GnRHa

2. Long acting progestogen versus GnRHa/ progestogen (injection)

3. Combined oral contraceptive pills versus placebo and progestogen

## Materials and methods

**Inclusion criteria**

Randomized controlled trials related to endometriosis-associated pain and medical treatments from the English literatures between 1995- 2009 were selected and pooled for analysis. 

**Exclusion criteria**

Cohort studies, case control and case reports were not considered. Studies which did not measure pain improvement as a measure outcome and did not match the above objectives were also not recruited. 

**Search**

Medline and Cochrane systemic review databases search using keywords: endometriosis, randomized controlled trial, pelvic pain, dysmenorrhea, combined oral contraceptive pills, progestagens and GnRHa were conducted. 

**Selection of studies**

Only medical treatments aimed at symptomatic improvement of pelvic pain were considered. Treatments with any progestogens, combined oral contraceptive pills, GnRHa and placebo were all considered, irrespective of dosage, route of administration or duration of treatment. Medical treatments for painful symptoms after conservative surgery were also considered in this review because of the paucity of randomized controlled studies. 

This analysis considered women of reproductive ages (18- 40 years) complaining of pain symptoms related to endometriosis. The endometriosis associated pain symptoms were: dysmenorrhoea, non-menstrual pelvic pain, chronic pelvic pain and deep dyspareunia. 

Studies where participants were asymptomatic or presented with infertility alone were not considered.

**Data extraction process**

This review included data from randomized controlled studies comparing control, progestogens, combined contraceptive pills and GnRHa in the treatment of endometriosis- associated pain. Two authors extract data independently concerning details of study design, study population, intervention and outcomes using a self-developed data extraction form. Any differences in data extraction were resolved by consensus, referring back to the original article. Any disagreement of data extraction was resolved by discussion with the senior academic author.

**Outcomes measures**

Outcome measures were considered at the end of treatment. The primary outcome measure was pain improvements for each pain symptoms where possible. Subjective pain relief measurement was considered using both visual analogue scale (VAS) and verbal rating scale (VRS). The occurrence of side effects was also considered as a secondary outcome measure.

**Outcome definitions**

It is defined that response to treatment was considered as a reduction in pain scores. Pain improvement was defined as improvement ≥1 at the end of treatment. 

Patient’s satisfaction with the treatment was considered if they were very satisfied or satisfied. 

**Statistical analysis**

Meta Analyst (6) Software was used in this project. Statistical analyses were performed to use the Relative Risk as the measure of effect for dichotomous data. 

There are many different existing methods to assess pain, standardized the mean difference were required. 

**Assessment of bias across studies**

We assessed the methodological quality using the standard as described by Kjaergard (2001) generation of the allocation sequence, allocation concealment, double blinding, and follow up (7). 

Based on these criteria, the risk of bias with all the features (random method, allocation concealment, blinding and follow up) of the studies was subdivided into the following three categories: all quality criteria met leading to low risk of bias; one or more of the quality criteria only partly met leading to moderate risk of bias; and one or more criteria not met leading to high risk of bias.

Jadad score was also used to assess the methodology quality of the clinical trial articles. A trial receives a score from zero to five. The evidence may be biased by selection bias, poor randomization and poor binding, which might affect the results of a trial. 

**Funding support**

There is no external funding support received on this project. 

## Results

**Study characteristics**

Seven articles (3, 4, 8-12) were recruited for further evaluation. Six (3, 4, 9- 12) out of seven studies were identified comparing progestogen versus other non-progestogen treatments. 

One study (8) compared the effectiveness of oral contraceptive pills versus control on relieving endometriosis associated pelvic pain. Another study (10) was evaluating the medical treatment in controlling the endometriosis-associated painful symptoms after conservative surgery. The main characteristics of studies were summarized in table I.

**Sample size**

A total of 1096 patients were recruited in seven studies. The sample size varied between studies. The mean numbers of patients included were 156 in the seven recruited randomized controlled trials. 

Endometriosis was staged according to the American Fertility Society classification in its original or revised form in three studies, while the remaining studies did not perform the staging of endometriosis.

**Measurement tools**

Majority of the studies used objective scales, such as verbal rating scale, a 10cm/100mm visual analogue scale and five rating scale, to assess the severity of pain. In two study (12, 13), patients were asked to rate their satisfactory level to the therapy; and treatment was considered beneficial if patients rated themselves as very satisfied or satisfied (12).

**Treatment schedule**

The mean duration of treatment was 7 months (range 3 to 12 months). 

Five recruited studies (3, 4, 9, 12, 13) used progestogen (depot medroxy-progesterone acetate, oral dinogest, implanon and levonorgestrel- releasing intrauterine system), four studied (3, 4, 9, 10) used GnRHa (Buserelin acetate, tryporelin, leuprerelin and lupron) and two studies (8, 10) used combined oral contraceptive pills (ethinyl- estradial 0.035mg+norethisterone 1mg; ethinyl estradiol 0.02 g, desogestrel 0.15 mg) as treatment intervention. 

**Risk of bias**

Three studies (4, 8, 10) (scored five in Jadad system which indicates having sufficient quality in the methodological quality assessment. 

Crosignani (2006) scored three in Jadad system because the method of blinding was not clearly stated (3). The evaluators were blinded but it was not specified if the patients were known to the medication that they were receiving. 

Another two studies (12, 13) also scored three and again there was no mention about the blinding. Petta (2005) scored two because there was unblended study with no clear description about dropout rate (9). 

**Progestogen versus GnRHa**

Progestogen (intrauterine device, DMPA and oral contraceptive pills) was compared with GnRHa in three of the seven randomized controlled trials (3, 4, 9). 

The three studies indicated prgestogens were as effective as GnRHa. They did not show to have a significant difference (Figure 2. RR: 0.036; CI -0.03, 0.102). 

In terms of side effects, GnRHa appeared to cause more bone mineral density loss than progestogens, therefore its use is usually limited to a period of 6 months. Treatment with progestogens was associated with a higher incidence of spotting. 

**Implanon versus Depot Medroxyprogesterone Acetate (DMPA)**

Implanon provides an alternative ways of delivering progestogen. Comparing long acting implanon and GnRHa/DMPA, there is no significant difference in relieving endometriosis-associated pain (Figure 3; RR: -0.006; CI: -0.142-0.162). 

Thus, the efficacy of implanon and Mirean is similar to that of GnRHa and DMPA in symptomatic endometriosis (13). Patients in both treatment groups experienced similar side effects such as weight gain, acne, loss of hair and breast tenderness. 

**Combined oral contraceptive pills versus control versus progestogen**

Combined oral contraceptive pills was compared with placebo in two randomized controlled trials and compared with DMPA (150 mg) in a randomized controlled trial. 

Combined oral contraceptive pills demonstrated effective treatment of endometriosis- associated pain when compared with placebo groups and reduced the use of analgesia (RR: 0.562; CI: 0.396-0.727). 

It also showed that oral contraceptive pills as an adjuvant therapy to surgery were more effective than surgery plus placebo to provide pain relief in patients with endometriosis stage 3- 4 (RR: 0.631; CI: 0.390-0.664).

Vercellini (1996) study showed that long acting progestogen is more effective than oral contraceptive pills in controlling dysmenorrhea despite all values in both groups were significantly reduced from baseline (RR: -0.160; CI: -0.386-0.066) (12). 

Progestogen is however associated with more spotting and bloating as side effects than the oral contraceptive pills. 

Overall, the meta- analysis of these studies showed that oral contraceptive pills is more effective to release pain than progestogen treatment (RR: 0.321; CI: -0.066-0.707).

**Table I:** Main characteristics of studies on the use of medical treatment of endometriosis-associated pain

**Reference**	**Type of study**	**Treatment schedule and follow-up **	**Sample size**	**Inclusive criteria**	**No. of women with pain at entry**	**Endometriosis stage**	**Criteria for pain evaluation**	**follow-ups**	**Jadad Score**
Harada *et al* (2008)^7^	A placebo-controlled double-blind randomized trial	Monophasic OCP (ethinyl-estradial 0.035mg + norethisterone 1mg) for 21 days plus 7 days of placebo, for 4 cycles	100	Moderate to severe dysmenorrhea	100	Not reported	Verbal rating scale (VRS) and visual analogue scale (VAS)	Response to treatment for dysmenorrhea	5
Harada *et al* (2009)^4^	A randomized double-blind, multicentre controlled trial	Dienogest (2mg/day, orally) or Buserelin acetate (900µg/day, intranasally) for 24 weeks	271	Non-menstrual Pelvic pain and objective findings (induration of the pouch of Douglas & limited uterine mobility)	271	Not reported	A five-level rating scale, VAS	Change in scores of subjective pain symptoms	5
Crosignani, Luciano, Ray and Bergqvist	A multicentre, evaluator-blinded comparator controlled randomized study	Medroxyprogesterone acetate (104mg/0.65ml, subcutaneously, every 3 months) or leuprolide (3.75mg monthly) for 6 months	300	Pelvic pain, Premenopausal women aged 18-49, with laparoscopic diagnosis	300	Not reported	Biberoglu and Behrman modified VRS scale: 0 (no discomfort) to 3 (severe pain)	Change in scores of subjective pain symptoms	3
Petta *et al* (2005)^8^	Multicentre randomized controlled clinical trial	Levonorgestrel-releasing intrauterine system (Mirena, 19-nortestosterone derivative); GnRH-analogue (Lupron depot 3.75mg) for 6 months	82	18-40 years old women, dysmenorrhoea, chronic pelvic pain	82	All stages	VAS	Improvement in endometriosis associated chronic pelvic pain and quality of life	2
Sesti *et al* (2007)^9^	A randomized comparative trial	1. Placebo or 2. GnRHa (tryporelin or leuprerelin, 3.75mg every 29 days) or 3. Continuous estoprogestin thynilestradiol 0.03mg plus gestoden 0.75mg) or 4. Dietary therapy (vitamins, minerals salts, lactic ferments, fish oil) for 6 months	222	Dysmenorrhea and/or non-menstrual pelvic pain and/or dyspareunia, up 40 years at the time of surgery	222	Stage 3 and 4 endometriosis (Revised AFS)	VAS	Improvement in pelvic pain and health-related quality of life	5
Walch *et al* (2009)^12^	An open, prospective. randomized, controlled clinical trial	Implanon (etonogestrel, subdermally) & depot medroxyprogesterone acetate (150mg, intramuscularly, every 3 months) for 12 months	41	Dysmenorrhoea, nonmenstrual pelvic pain and dyspareunia	41	All stages (Revised AFS)	A 100mm VAS	Change in pain score after 6 months Follow up every 3 months for 1 year	3
Vercellini^2^ *et al* (1996)^11^	A randomized clinical trial	DMPA (150mg, intramuscularly) vs oral contraceptive (ethinyl estradiol 0.02g, desogestrel 0.15mg) + danazol (50mg a day for 21 days of each 28 day cycle) for 1 year	80	18-40 years old women, moderate to severe pelvic pain, Pelvic pain > 6 months	80	All stages (Revised AFS)	A 10 cm visual analogue scale	Improvement in pain symptoms and patients’ satisfaction at the end of therapy	3

**Figure 1:**
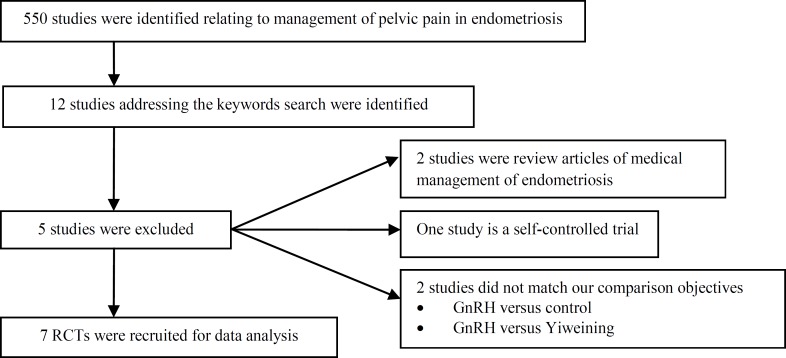
A flow chart demonstrates the identification, recruitment and exclusion of studies

**Figure 2:**
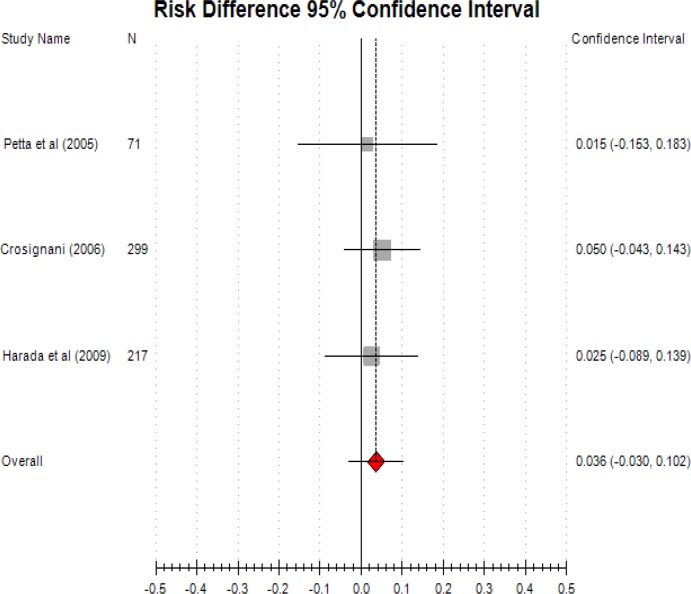
Progestogen versus GnRHa

**Figure 3:**
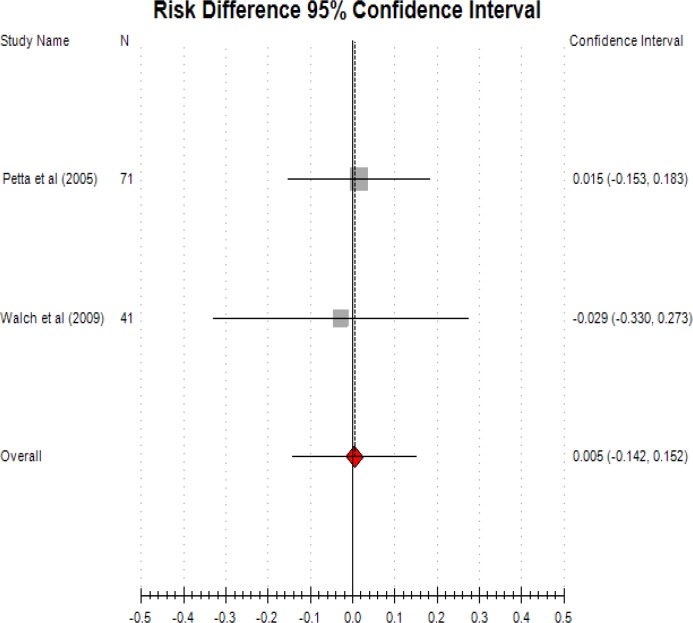
LNG-IUS versus GnRHa and Implanon (etonogestrel) versus DMPA.

**Figure 4:**
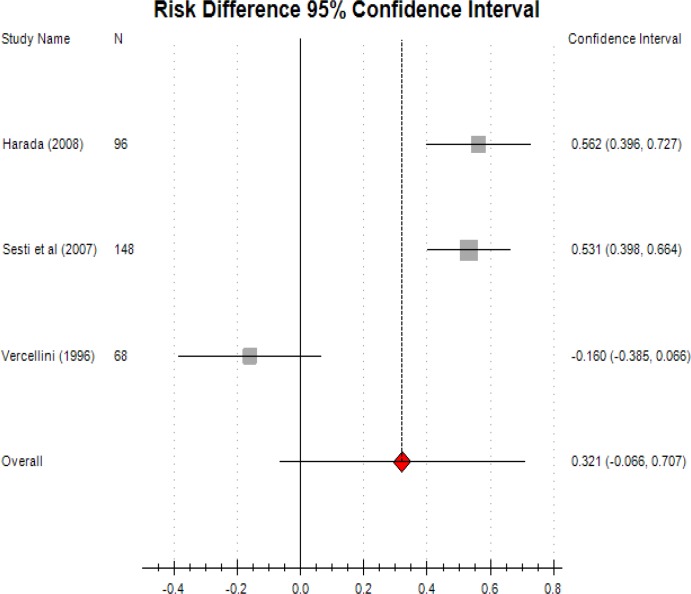
Combined oral contraceptive pills versus placebo versus progestogens

## Discussion

There is a paucity of randomized controlled trials in relating to endometriosis associated pain symptoms. Disease staging was not always uniformly employed in the studies, which limited the evaluation of the severity of pain against the effectiveness of medical treatment. 

Furthermore, small sample sizes in some studies (9, 12, 13) limited the ability to draw definite conclusions. Overall, the results from our analysis of pooled data from available randomized controlled studies in the English literature suggest that progestogens and long acting progestogen might have slightly better result that GnRHa, but oral contraceptive pills have a higher level of efficacy than progestogen.

The seven articles demonstrated a significant reduction in pain scores after the commencement of medical treatments. Five articles used Visual analogue scale (VAS) to measure the severity of pain pre- and post- treatment. VAS is a widely used pain assessment tool, which provides a continuous scale for subjective rating along the line. The extremes carry a verbal description of symptoms to be evaluated such as the most severe pain and no pain. The advantages of using VAS are time-saving and cross-culture. However, Langley and Sheppeard (1985) question the validity of VAS measurements due to its propensity to bias (14). 

Firstly, the physical characteristics of the scale might affect the accuracy of the scale. “No pain” is influenced by subjective individual pain threshold and some patients might have difficulties in distinguishing discomfort and pain. Likewise, “the most severe pain” is an infinite description. It can be influenced by personal experience. Also, patients’ behavior when completing the scale might lead to bias. They tend to recall memory of the previous pain scores. This might influence the accuracy of the pain measurement (14). 

One study included in this review used verbal rating scale (VRS) which consists of a set of descriptive words. A study comparing VRS and VAS showed that the pain scores in the middle are liner-related but not the upper and lower extremes (15). Thus, there exists an inherent discrepancy in pain measurements, due to the subjectivity of the pain experience. Pain is influenced by multiple factors such as personal belief, culture, past experience and emotion, it is difficult to assess pain as a whole. Nevertheless, VRS and VAS are useful to measure the intensity of pain in short term despite considerable uncertainty regarding their long- term use as serial measurements.

Comparisons of progestogens and GnRHa have been made. Two of the three studies have a relatively large sample sizes, both proved that progestoegn is as effective as GnRHa, although neither treatment appears superior (3, 4). Apart from looking at short-acting progestogen such as oral and depot, we have included other long-acting progestogen in treatment in endometriosis associated pain. Mirena, the intrauterine device, releases levonorgestrel directly into the uterine cavity at a relative constant rate of 20 µg/ day for 5 years. Although its mechanism is still unclear, it has been speculated that progestogen induced endometrial atropy leading to amenorrhea (9). Petta (2005) demonstrated the short term effect (6 months) of Mirena in controlling endometriosis-associated pain (9). Since the release of levonorgestrel may slowly reduce in the 5 years of use, there is no evidence showing the efficacy of Mirena in controlling endometriosis-associated pain in long- term. Additionally, the longer the effect of Mirena in pain control, the more cost-effective it will be. 

Implanon is a single rod etonogestrel- containing contraceptive implant. It is inserted subdermally and provides a slow release of progestogen. It lasts for three years. Efficacy of Implanon is not inferior to DMPA. A single insertion of implanon is more convenient than an injection every 3 months. Both Mirena and Implanon are long- term treatment options in women with symptomatic endometriosis who also require contraception. 

Complications and withdrawal of treatments are other measures to look at the overall effectiveness of the treatments. In some studies, side effects were only considered if they were severe enough to cause withdrawal of the patient. Many patients on progestogen treatments experienced side effects such as irregular bleeding, bloating and weight gain. 

Women on GnRHa complained of hot flushes severe enough to stop treatment. Additionally, GnRHa causes loss of bone mineral density which limits its long-term use. Despite high reporting rate of side effects, there was a relatively low dropout rate in these studies. This could indicate that the presence of the side effects could be well tolerated. It is questionable whether the severity of side effects significantly increased dropout rates, impacting an overall effectiveness.

Compliance is always an issue in medical management. Combined pills and oral progestogens were taken everyday. The studies did not measure the rate of compliance. Moreover, it was often unclear if the recruited patients were taking alternative medications, which may have a significant confounding variable affecting outcomes.

Follow- up is an important factor in monitoring a chronic disease with high probability of recurrence. Follow- up data in different studies were referred to different lengths. Most studies could not prove effectiveness in long- term management of endometriosis-associated pain since it is common to have symptoms recurrence once the treatment stops. Similar with surgical intervention, there is always a chance of relapse. 

## Conclusion

It appears that combined oral contraceptive pills, GnRHa and progestogens are all effective and well tolerated by patients in treating endometriosis associated pain, though side effects have to be considered. Combined oral contraceptive pills and progestogens are relatively cheap and more suitable for long-term use as compared to GnRHa. Even though both Mirena and Implanon had a role in controlling endometriosis pain, the conclusion was drawn from a study with relatively small sample sizes. Longer term follow up studies of Mirena and Implanon are required to look at their long-term effects on endometriosis associated pain. 
